# Unaddressed privacy risks in accredited health and wellness apps: a cross-sectional systematic assessment

**DOI:** 10.1186/s12916-015-0444-y

**Published:** 2015-09-07

**Authors:** Kit Huckvale, José Tomás Prieto, Myra Tilney, Pierre-Jean Benghozi, Josip Car

**Affiliations:** Global eHealth Unit, Imperial College London, Reynolds Building, St Dunstan’s Road, London, W6 8RP UK; CRG, Ecole polytechnique CNRS, Bâtiment Ensta, 828 boulevard des Maréchaux, Palaiseau, Cedex 91762 France; Health Services and Outcomes Research Programme, LKC Medicine, Imperial College, Nanyang Technological University, Singapore, Singapore

**Keywords:** Smartphone, Mobile, Apps, Accreditation, NHS, Privacy, Confidentiality, Cross-sectional study, Systematic assessment

## Abstract

**Background:**

Poor information privacy practices have been identified in health apps. Medical app accreditation programs offer a mechanism for assuring the quality of apps; however, little is known about their ability to control information privacy risks. We aimed to assess the extent to which already-certified apps complied with data protection principles mandated by the largest national accreditation program.

**Methods:**

Cross-sectional, systematic, 6-month assessment of 79 apps certified as clinically safe and trustworthy by the UK NHS Health Apps Library. Protocol-based testing was used to characterize personal information collection, local-device storage and information transmission. Observed information handling practices were compared against privacy policy commitments.

**Results:**

The study revealed that 89 % (n = 70/79) of apps transmitted information to online services. No app encrypted personal information stored locally. Furthermore, 66 % (23/35) of apps sending identifying information over the Internet did not use encryption and 20 % (7/35) did not have a privacy policy. Overall, 67 % (53/79) of apps had some form of privacy policy. No app collected or transmitted information that a policy explicitly stated it would not; however, 78 % (38/49) of information-transmitting apps with a policy did not describe the nature of personal information included in transmissions. Four apps sent both identifying and health information without encryption. Although the study was not designed to examine data handling after transmission to online services, security problems appeared to place users at risk of data theft in two cases.

**Conclusions:**

Systematic gaps in compliance with data protection principles in accredited health apps question whether certification programs relying substantially on developer disclosures can provide a trusted resource for patients and clinicians. Accreditation programs should, as a minimum, provide consistent and reliable warnings about possible threats and, ideally, require publishers to rectify vulnerabilities before apps are released.

**Electronic supplementary material:**

The online version of this article (doi:10.1186/s12916-015-0444-y) contains supplementary material, which is available to authorized users.

## Background

Mobile apps – software programs running on devices like smartphones – offer a wide range of potential medical and health-related uses [1]. They are part of a consumer phenomenon which has seen rapid uptake and evolution of mobile hardware and software. Market estimates suggest that almost half a billion smartphone users worldwide use a health or wellness app, a figure that is set to treble in the next 3 years [2]. In consumer surveys, a quarter of US adults report using one or more health tracking apps and a third of physicians have recommended an app to a patient in the past year [3, 4]. As apps offering monitoring and self-management functions become commonplace, the opportunities for collecting and sharing personal and health-related information will grow. These changes bring potential clinical benefits [5], but also expose new risks [6].

Because users cannot see into the inner workings of apps, or the services they connect to, confidence that personal information is handled appropriately relies mostly on trust. Users must trust in the ethical operation of app services, that developers will comply with privacy regulation and security-best practices, and that app marketplaces and regulators will intervene, if necessary, to safeguard user interests. Health apps may put patient privacy, defined here as the right of individuals to control how their information is collected and used [7] (other definitions are possible [8]), at risk in a number of ways [9]. Medical information stored on devices that are lost or stolen may be accessed by malicious users, particularly if information is not secured using encryption. Information may be shared unexpectedly because privacy practices and settings are confusing or poorly described. Some apps may offer free services in return for access to personal information, an arrangement to which users can only give informed consent if fully disclosed. When physical, technical or organizational confidentiality arrangements are inadequate, information transmitted online may be at risk of interception or disclosure [7]. Global computer networks make it easy for personal information to be transferred, inadvertently or otherwise, into jurisdictions with reduced privacy protections. Recent privacy-focused reviews of health and wellness apps available through generic app stores have consistently identified gaps in the extent to which data uses are fully documented and appropriate security mechanisms implemented [10–13].

Medical app accreditation programs, in which apps are subject to formal assessment or peer review, are a recent development that aims to provide clinical assurances about quality and safety, foster trust, and promote app adoption by patients and professionals [14–18]. Privacy badging of websites has been found to lead to modest benefits in the extent to which information uses and security arrangements are openly disclosed [19]. However, the privacy assurances offered by app programs are largely untested. In late 2013, one service had to suspend its approval program after some apps were found to be transmitting personal information without encryption [20]. We aimed to understand if this was an isolated example or symptomatic of more systematic problems in controlling privacy-related risks. We used a systematic method to appraise all health and wellness apps approved by a prominent national accreditation program; the English National Health Service (NHS) Health Apps Library [14].

Launched in March 2013, the NHS Health Apps Library offers a curated list of apps for patient and public use. Apps are intended to be suitable for professional recommendation to patients but are also available for general use without clinical support. Registered apps undergo an appraisal process, developed in response to concerns raised by UK healthcare professionals [21, 22], that aims to ensure clinical safety and compliance with data protection law. In the UK, the major governing legislation is the Data Protection Act 1998 [23]. This enshrines eight data protection principles which place limits on the appropriate and proportionate collection and use of personal information, require that these uses are clearly specified (for example, in a privacy policy), establish the rights of individuals to control and amend their information, and mandate safeguards against situations that might compromise these rights, such as unauthorized access to data. In respect of privacy, the accreditation approach adopted by the Health Apps Library is to require developers to declare any data transmissions made by their app and, in this case, to provide evidence of registration with the Information Commissioner’s Office (ICO), the UK body responsible for enforcement of the Data Protection Act (information obtained via Freedom of Information request). Registration entails a commitment to uphold principles of data protection and is a requirement under the Act for individuals or organizations processing personal information. Thus, while substantially relying on self-declaration, it is clear the expected intent, and the assumption that might reasonably be made by app users, is that apps accredited by the NHS Health Apps Library will comply with UK data protection principles concerning information privacy.

The purpose of the current study was to assess the extent to which accredited apps adhered to these principles. We reviewed all apps available from the NHS Health Apps Library at a particular point in time, and assessed compliance with recommended practice for information collection, transmission and mobile-device storage; confidentiality arrangements in apps and developer-provided online services; the availability and content of privacy policies; and the agreement between policies and observed behaviour.

## Methods

### App selection

All mobile apps available in the NHS Health Apps Library in July 2013 and targeting Android and iOS, the two most widely-used mobile operating systems, were eligible for inclusion. Apps were excluded if they could not be downloaded or cost more than 50 USD. Free, demo and ‘lite’ apps were excluded if a full version was available. Apps were also excluded if they would not start, after two attempts on different test devices. Apps available on both Android and iOS platforms were downloaded and evaluated separately, but combined for analysis. To ensure consistency, no app or operating system updates were applied during the evaluation period.

### Overview of assessment approach

Assessment involved a combination of manual testing and policy review. Testing was used to characterize app features, explore data collection and transmission behaviour, and identify adherence to data protection principles concerning information security. Policy review identified the extent to which app developers addressed data protection principles concerning disclosure of data uses and user rights. In a final step, policy commitments were compared and contrasted with behaviours actually observed in apps. These processes are described further below.

### App testing

Apps were subject to a 6-month period of evaluation, from August 2013 to January 2014. Testing incorporated two strategies. To ensure coverage of features relating to information collection and transmission, sequential exploration of all user interface elements was performed for each app. After this, apps were subject to an extended period of testing which included periods of both routine daily use and less frequent, intermittent interaction. The aim of this extended process was to uncover app behaviours that might occur only infrequently but were relevant from a privacy point of view, for example time-delayed solicitation of feedback or transmission of aggregated analytics data.

Testing was performed by two study researchers, working independently. If required, simulated user information was used to populate user interface elements. For apps that required user accounts, app-specific credentials were generated. Diaries and logs were completed by supplying clinically-plausible information. In a small number of cases (n = 2), apps offered mechanisms to request support related to the app purpose, such as a telephone call-back to receive more information about a particular service. A further six apps incorporated user experience feedback forms. To avoid misleading potential recipients of either support requests or feedback, we annotated simulated information used to exercise these functions to indicate its status as a test communication that should be discarded and not acted upon. Recognizing that such flagging might act as a potential source of bias if data handling were altered as a result, these activities were performed at the conclusion of the testing process and once other aspects of app behaviour, data collection and transmission had been characterized. Because the study involved only simulated user data, and involved no human participants, informed consent was not required.

### Data entry and mobile-device storage assessment

Types of data that could be entered through the user interface of each app were coded (data types listed in Additional file 1: Table AF1). At the conclusion of the evaluation period, data stored on each test device were transferred to a research computer for examination. File management software [24, 25] was used to copy user data files and app-specific data caches. Files were inspected to catalogue the types of content being stored for each app, and to identify any mechanisms used to secure data, for example encryption. Because assessment was not performed with the involvement of developers, we only had access to storage at the device level, and were unable to assess data stored in online services.

### Data transmission assessment

**Fig. 1:**
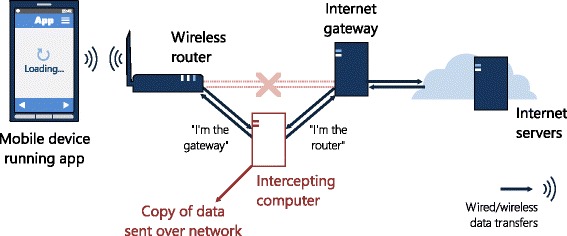
A ‘man-in-the-middle’ attack. A man-in-the-middle attack is able to intercept network traffic sent by a mobile app in a way that is invisible to users and services

To capture data transmitted by included apps, we reconfigured local network infrastructure so that a copy could be obtained without interrupting the flow of communication, a form of eavesdropping known as a ‘man-in-the-middle’ attack (Fig. 1) [26]. The advantage of this approach was that it required no modification to either apps or online services that might have affected the process of data exchange and bias interpretation. We combined an existing open source software tool [27] with custom scripting and back-end database to capture and store all traffic generated during the test period. By making a simple configuration change to the operating systems on each test device, we were able to intercept encrypted communications in addition to unsecured traffic (principles explained in Additional file 1: Figure AF2) [28].

Prior to the start of the evaluation, we conducted pilot testing using a range of system and user apps not included in the study to ensure that all data would be captured. We anticipated that some test apps might implement certificate pinning [29], a technical security measure designed to prevent man-in-the-middle attacks on encrypted communications. However, in practice, this was only observed for certain communications generated by the mobile operating system and did not affect interception of traffic generated by test apps.

Personal information sent by apps was categorized in a two-part process, using the same coding schema used to analyze data collection (Additional file 1: Table AF1). In the first step, an automated process was used to classify data according to destination and the mechanisms used to secure the content, if at all. Known instances of particular data types were also identified automatically by searching for user details generated during testing such as app-specific simulated email addresses. No data were discarded during automatic coding. In the second step, the content of captured traffic was displayed in a custom software tool for manual review (see Additional file 1: Figure AF3). Although all traffic was inspected, multiple transmissions with identical content (excluding timestamps) were automatically combined for review. The review process allowed study reviewers to check automatic tagging and manually code any personal information not already identified. Coding was performed by two researchers, working independently, and reconciled through discussion.

Potential vulnerabilities in server-side controls were explored through manual testing. To identify potential authorization problems, we reset session state information and replayed intercepted data requests to see if developer or third-party systems would return user data without first requiring suitable authorization. Manual inspection of transmission also identified one instance where messages incorporated parameterized database queries potentially susceptible to malicious modification through SQL injection. To confirm this possibility, we modified an existing request to return data associated with a second, simulated user account. During vulnerability testing we did not attempt to modify or delete data, nor did we access data belonging to user accounts not under our control.

### Policy review

We systematically assessed the content of privacy policies associated with each app. We searched in-app documentation, app store entries and related websites. All documents that self-identified as a privacy policy were included in analysis. We also located privacy-relevant text forming part of other policy documents, for example terms and conditions, disclaimers, and app-store marketing text. We developed a coding schema based on guidance produced by the UK ICO [30] concerning requirements established by the Data Protection Act 1998 (see Additional file 1: Table AF4). The schema was used to assess coverage of data protection principles, for example disclosure of primary and secondary uses of information, the intent to physical information confidentiality measures, and mechanisms for accessing, modifying and deleting personal information.

Assessment proceeded by systematically coding each schema item as either being addressed or absent from extracted policy text. For those principles relating to user rights, such as the ability to opt-out of data collection, policies were additionally coded according to whether a given right was afforded or denied in policy text. However, while there were multiple instances where policies made no reference to certain rights, there were no instances where a user right was mentioned only to be denied. Separately, policies were coded using the data item schema (Additional file 1: Table AF1) to identify the extent to which policies provided a complete and correct account of those data items being transmitted to a developer or third party. Individual data items were coded as either ‘will transmit’, ‘will not transmit’ or ‘not addressed by policy’. Coding was performed by two researchers, working independently.

Coding decisions, as well as any relevant policy text annotations, were captured using custom software (see Additional file 1: Figure AF5). All decisions were reviewed to reach a consensus agreement on policy coverage. The nature of information actually collected and transmitted by apps was then compared to specific commitments made in privacy policies. We also recorded the operating system permissions requested by each app at installation or during subsequent use, for example access to user contacts or geolocation service, as well as configuration options offered by each app to control the transmission of data to developer and third-party services.

### Statistical analysis

Data were compiled into a single dataset for analysis (supplied as Additional file 2). We used simple descriptive statistics to summarize aspects of data collection, mobile-device storage and transmission. Unless otherwise stated, the unit of analysis is the platform-independent app. Expectations that apps available on both iOS and Android would substantially share privacy-related characteristics were confirmed. Therefore, to avoid double counting, we combined these apps for analysis. Because of the potential risk to current users, we have chosen not to identify specific apps with confidentiality vulnerabilities. However, in November 2014, the NHS Health Apps Library was provided with details of the vulnerabilities we identified.

We hypothesized that the likelihood of an app having a privacy policy should not vary by platform or by the distribution model (free or paid). Although apps for iOS are subject to a quality control process prior to release, and although developers of paid-for apps may have greater resources to invest in quality assurance, an accreditation program should apply the same privacy evaluation rules to apps, and no significant differences should be found. We used Fisher’s exact test to calculate the two-tailed probability of an association between the transmission of personal information and platform, distribution model and availability of a privacy policy. Statistical analysis was done in STATA, version 10.0 for Macintosh. We recognized that the type of app might differ by cost and payment model, and that this might confound analysis by altering the requirement for a privacy policy. For example, information-only apps might not require a policy, and might also be more likely to be freely available. Consequently, we performed a simple sensitivity analysis by considering only those apps that collected or transmitted personal or sensitive information. A significance level of 0.05 was pre-specified for all comparisons.

### Ethics statement

We used the Imperial College Research Ethics Committee process flow chart (version 22/05/2014) and the Medical Research Council (MRC) NHS Health Research Authority Decision Tool (not versioned) to establish that ethics approval was not required for this study, which did not involve human participants or data pertaining to individuals, and was conducted without involving NHS staff or facilities.

## Results

### App characteristics

In July 2013, the NHS Health Apps Library contained 88 apps. After excluding 9 (details in Additional file 1: Table AF6), 79 were selected for assessment (listed in Additional file 1: Table AF7). Basic characteristics of included apps are summarized in Table 1. Nearly half (48 %, n = 38/79) of apps were available for both iOS and Android, 47 % (n = 37/79) were iOS-only, and 5 % (n = 4/79) were Android-only.

**Table 1:** Basic characteristics of included apps

Characteristic	Apps with characteristic, n = 79 (%)
Platform	
Both platforms	38 (48 %)
iOS-only	37 (47 %)
Android-only	4 (5 %)
Cost	
Free	58 (73 %)
Paid-for	21 (27 %)
Version history	
First releases	17 (22 %)
Older	62 (78 %)
App purposes^a^	
Information provision	46 (58 %)
Healthy living and health promotion	21 (27 %)
Exercise and weight loss	8 (10 %)
Smoking cessation	6 (8 %)
Alcohol use	8 (10 %)
Drug use	5 (6 %)
Sexual health	5 (6 %)
Self-management	16 (20 %)
Long-term conditions	10 (13 %)
Diabetes	5 (6 %)
Hypertension	2 (3 %)
Other^b^	5 (6 %)
Therapy-related management	11 (14 %)
Self-assessment	9 (11 %)
Diary or personal health record	27 (34 %)
Medication management	3 (4 %)
Reminders	4 (5 %)
Assistive technologies	4 (5 %)
Service directory or finder	9 (11 %)
Social networking	4 (5 %)
Collecting data for research	6 (8 %)

^a^Most apps supported multiple functions and are counted more than once; ^b^epilepsy (n = 1), irritable bowel syndrome (n = 1), Parkinson’s disease (n = 1), sickle cell anemia (n = 1), stroke (n = 1)

Included apps addressed a wide range of health and health-related functions. Almost two-thirds of apps (58 %, n = 46/79) offered written or multimedia information. A quarter (27 %, n = 21/79) addressed health promotion topics such as weight loss (10 %, n = 8/79) and alcohol harm reduction (10 %, n = 8/79). A fifth (20 %, n = 16/79) of apps provided tools to assist aspects of either long-term condition self-care (11 %, n = 9/79) or self-management relating to particular therapies and procedures (14 %, n = 11/79), for example perioperative care or chemotherapy side-effect surveillance. One in ten (11 %, n = 9/79) of apps offered a self-assessment function. These included providing advice based on symptoms (n = 4), calculating future disease-related risk (n = 4) for conditions including breast cancer and cardiovascular disease, and testing eyesight (n = 1). A third of all apps (30 %, n = 24/79) provided some form of diary to record condition or health-specific information, for example medical history details or blood glucose measurements. Several apps provided assistive functions for those with speech and language (n = 3) and motor (n = 1) impairments, for example text-to-speech synthesizers. Nine apps provided access to location-based listings of health services. One in seven apps (15 %, n = 12/79) were NHS-branded products released in association with the UK Department of Health or local NHS bodies.

### Results of data entry and mobile-device storage assessment

Most apps (89 %, n = 70/79) included some form of data entry mechanism. The majority of these (83 %, n = 58/70) collected generic user-generated content such as annotations (20 %, n = 14/70), appointments (14 %, n = 10/70), bookmarks (16 %, n = 11/70), feedback ratings (17 %, n = 12/70), messages (7 %, n = 5/70), reminders (19 %, n = 13/70) and diaries (6 %, n = 4/70). However, two-thirds (64 %, n = 45/70) allowed users to enter strong identifiers, most commonly email addresses (31 %, n = 22/70), username/password information (30 %, n = 21/70) or a full name (23 %, n = 16/70). A fifth (20 %, n = 14/70) of apps included a registration process involving the creation of a user account. Registration was mandatory in most (71 %, n = 10/ 14) of these apps. Eight apps (11 %, n = 8/70) allowed photographs of people to be stored, for example as a user avatar or to track progress in weight loss. Half (49 %, n = 34/70) captured weaker identifiers, for example gender (26 %, n = 18/70), ‘blurred’ [31] location data, for example partial postcode (20 %, n = 14/70), or age (16 %, n = 11/70). Almost three-fifths (57 %, n = 40/70) of apps captured potentially sensitive information. Most (56 %, n = 39/70) recorded health-related details consisting of measurements made by users (n = 6), other medical history details (n = 14), or both (n = 13). Reflecting the number of apps (27 %, n = 21/79) addressing health promotion topics, almost a fifth of apps (19 %, n = 13/70) captured information relating to alcohol, smoking and substance use. A small proportion of apps (7 %, n = 5/70) captured other sensitive information, including ethnicity (n = 3), employment status (n = 2) and sexuality (n = 1). The majority of apps (84 %, n = 59/70) also collected other types of user-generated content. This information was commonly related to aspects of health self-management, for example annotations in personal health records (20 %, n = 14/70), reminders (19 %, n = 13/70) or appointments (14 %, n = 10/70). Some apps collected information explicitly intended to be shared with others. Both service directories and some health promotion apps included features for users to share opinions and feedback on services and content (16 %, n = 11/70). A small number of apps (6 %, n = 4/70) allowed users to post messages to a forum or to other users.

Most apps accepting user-entered information (96 %, n = 67/70) stored information on the device. Although over half (53 %, n = 42/79) of apps stored personal or sensitive information, no app encrypted local data stores (Table 2). Of the 21 apps offering a username-password combination or PIN to secure information, three-fifths (62 %, n = 13/21) took steps to protect the information by either not saving details locally (n = 10) or using a hash or token instead (n = 3). However, two apps revealed credentials anyway because the details had been captured in temporary files kept by the device, a phenomenon known as leakage [26]. Consequently, account details were easily accessible in over half of apps (52 %, n = 11/21). Of the three apps which did not save information, two were self-assessment questionnaires designed to calculate a disease risk score and one was a social network where all data were hosted online.

**Table 2:** Security vulnerabilities affecting data storage and transmission

Security vulnerability class [49]	Type	All apps, n = 79 (%)
Insecure data storage	Unencrypted data storage (of any data)	73 (92 %)
	Unencrypted username/password	8 (10 %)
	Unencrypted personal or sensitive information^a^	42 (53 %)
Insufficient transport layer protection	Identifying information sent without encryption^b^	23 (29 %)
	Sensitive information sent without encryption	6 (8 %)
Unintended data leakage	Username/password captured in network cache or log	2 (3 %)
	Health-related information sent to third parties	8 (10 %)
	Fixed device identifier used as user identifier	9 (11 %)
Weak server-side controls	Unencrypted access to server-side API	16 (20 %)
	Access to user data without authorization	2 (3 %)

^a^Excluding username and password; ^b^considering strong identifiers only

### Results of data transmission assessment

The majority of apps (89 %, n = 70/79) communicated with online services. Of those apps that transmitted information, a third (33 %, n = 23/70) sent information to a developer-controlled server (Additional file 1: Table AF8). Most (90 %, n = 63/70) also communicated with one or more third-party services directly. The most common reason for information transmission (in 87 %, n = 61/70) involved loading content from a developer (n = 8) or third-party (n = 49) service, or both (n = 4), in response to specific requests generated by a user, for example searching for specific health information. Nine apps sent user account details and other user-generated information to a developer (n = 7) or third-party service (n = 2) to provide services like account registration or to enable information to be accessed on multiple devices. Six collected crowd-sourced user feedback, for example anonymous ratings of local health services. Two-thirds of apps collected information for either analytics intended to assist product development and improvement (61 %, n = 43/70), or as part of a specific research project (9 %, n = 7/79). While only six apps included visible advertising, for example banner adverts, just under a fifth of apps (17 %, n = 14/79) sent information to advertisers or marketers directly. Marketing and advertising-related transmissions most commonly occurred because of tracking code included in web-based content that was downloaded and displayed within apps, rather than due to tracking features of the apps themselves.

Three-quarters of apps that transmitted information (74 %, n = 52/70) sent information to locations outside the UK, including the USA, Australia, Germany, Ireland and the Netherlands. All were countries recognized by the European Commission as providing adequate levels of data protection [32]. Analytics and marketing services were those most likely to be hosted overseas (Additional file 1: Table AF8). Nearly all apps sending analytics data (98 %, n = 42 of 43), and two-thirds of those sending marketing data (64 %, n = 9/14), did so to non-UK servers.

Half of apps (50 %, n = 35/70) included strong identifiers in transmitted information. Two-thirds of these (66 %, n = 23/35, 29 % of all apps, Table 2) sent identifying information without encryption, including email addresses (n = 5), account login details (n = 5), full name (n = 2) or date of birth (n = 2). Twelve apps (17 %, n = 12/70) transmitted health-related information (n = 7), other sensitive information (n = 3), or both (n = 2). Half (n = 6) encrypted transmission of this information. Of the remaining six which did not encrypt information, two were research projects which paired information about weight (n = 1) and substance use (n = 1) with weaker identifiers. Four apps, however, sent health-related information together with strong personal identifiers to a developer-hosted (n = 2) or third-party cloud service (n = 2) without encryption. Six apps sent either strong identifiers (n = 3), sensitive information (n = 1), or both (n = 2), to servers hosted outside the UK without encryption. No association was found between payment model or platform and the transmission of personal information.

No app deliberately sent strong identifiers or sensitive information to advertisers, marketing companies or other content providers. A small number of apps risked leaking information about user health status by including details of search queries or page views in information provided to advertisers (n = 5) or other third parties (n = 3); however, this information was not accompanied by user-supplied identifying details. Instead, advertisers routinely generated their own identifiers or cookies which were then stored and used by apps in lieu of other identifiers to track usage. Transmissions to analytics services commonly used a similar mechanism to identify users. However, 21 % (n = 9/43) of apps communicating with these services used a fixed device identifier, rather than a randomly generated key, to identify users. Only one app offered an in-app mechanism for disabling transmission to analytics or third-party services.

In addition to issues affecting information transmission, risks to confidentiality were identified in software application programming interfaces (APIs) provided by online services to communicate with apps. Of 27 apps with such services, 16 (59 %, n = 16/27) allowed unencrypted access. Two apps had critical vulnerabilities which permitted access to user information, including information belonging to other users, without authorization. The first did not implement any form of access control, and the second was susceptible to SQL injection, a type of attack that manipulates queries sent to a database [33].

### Results of policy assessment

Two-thirds (67 %, n = 53/79) of apps had some form of privacy policy (Table 3). The proportion was higher in apps in which users could record information (71 %, n = 50/70) and those transmitting user-related information (70 %, n = 49/70). The availability of a privacy policy differed by payment model (*P* = 0.015) but not by platform (*P* >0.99). Three-quarters (74 %, n = 43/58) of free apps had a disclosure versus two-fifths (43 %, n = 9/21) of paid-for apps. Considering apps only available on a single platform, two-fifths (62 %, n = 23/37) of iOS apps had a disclosure versus three-quarters (75 %, n = 3/4) of Android apps.

**Table 3:** Availability of policy disclosures

		Apps collecting data		Apps transmitting data	
Policy	All apps, n = 79 (%)	Any data, n = 70 (%)	Personal or sensitive data^a^, n = 59 (%)	Any data, n = 70 (%)	Personal or sensitive data^a^, n = 38 (%)
Privacy disclosure available	53 (67 %)	50 (71 %)	43 (73 %)	49 (70 %)	31 (82 %)
In-app privacy policy	22 (28 %)	22 (31 %)	21 (36 %)	22 (31 %)	15 (39 %)
Other privacy policy	48 (61 %)	45 (64 %)	38 (64 %)	44 (63 %)	29 (76 %)
Policy mentions app	8 (10 %)	8 (11 %)	5 (8 %)	8 (11 %)	5 (13 %)
Advertising policy	3 (4 %)	3 (4 %)	3 (5 %)	3 (4 %)	3 (8 %)
No privacy disclosure	26 (33 %)	20 (29 %)	16 (27 %)	21 (30 %)	7 (18 %)
In-app clinical disclaimer	36 (46 %)	32 (46 %)	26 (44 %)	33 (47 %)	13 (34 %)

^a^Incorporates strong personal identifiers, health-related information and other sensitive information

Approximately a third (35 %, n = 28/79) of all apps, and two-fifths (42 %, n = 25/59) of those collecting personal or sensitive data, were linked to some form of app-specific policy. Less than a third of apps (28 %, n = 22/79) incorporated a privacy policy within the app itself. A small number of apps provided links to external policies that did not work (n = 4), and one app crashed repeatedly when trying to view the policy. Three-fifths of apps (61 %, n = 48/79) provided a disclosure on an associated website, but only 10 % (n = 8/79) of these web disclosures specifically referred to the app. A small number of apps (4 %, n = 3/79) had a separate advertising policy. Use of just-in-time privacy notifications was restricted to prompts generated by the underlying operating systems when contacts, notifications or location permissions were requested.

Policies varied in their coverage of recommended topics (Table 4). Most apps (87 %, n = 46 of 53 apps with policies) incorporated statements of intended primary uses for recorded or transmitted information, and three-fifths (58 %, n = 31/53) described intended secondary uses. The extent to which these uses corresponded to collected and transmitted data is discussed further below (see Concordance of policies and data handling practices). Because we did not observe developer practices, the possibility that these were only partial accounts of actual uses cannot be excluded. Coverage of other privacy domains was more variable. Perhaps reflecting the proportion of policies sourced from the Internet, four-fifths (79 %, n = 42/53) described how cookies would be used, but only slightly more than half (53 %, n = 28/53) discussed data confidentiality mechanisms, and fewer than one-fifth (17 %, n = 9/53) addressed data retention. Three-fifths of apps which transmitted information explained how to edit specific information held by developers or third parties (55 %, n = 29/53), or to opt-out of aspects of information sharing (57 %, n = 30/53), but less than a third offered a mechanism for viewing all information (26 %, n = 14/53) or requesting its deletion (28 %, n = 15/53). While half (51 %, n = 27/53) of apps indicated the jurisdiction governing the policy, a small proportion clearly identified the legal entity responsible for the policy and any data (30 %, n = 16/53), or the jurisdictions under which data would be processed (25 %, n = 13/53).

**Table 4:** Coverage of privacy and security-related topics in privacy policies

		Apps with a privacy policy				
			Apps collecting data		Apps transmitting data	
Domain	Topic	All apps, n = 53 (%)	Any data, n = 50 (%)	Personal or sensitive data^a^, n = 43 (%)	Any data, n = 49 (%)	Personal or sensitive data^a^, n = 31 (%)
Uses of data	Primary uses of collected data	46 (87 %)	43 (86 %)	36 (84 %)	43 (88 %)	28 (90 %)
	Secondary uses of collected data	31 (58 %)	29 (58 %)	25 (58 %)	30 (61 %)	20 (65 %)
	Sending data to developer-provided online services	21 (40 %)	21 (42 %)	18 (42 %)	21 (43 %)	17 (55 %)
	Sending data to advertisers/marketers	6 (11 %)	6 (12 %)	6 (14 %)	6 (12 %)	6 (19 %)
	Sending data for analytics/research	19 (36 %)	18 (36 %)	14 (33 %)	19 (39 %)	16 (52 %)
	Sending data while loading content	5 (9 %)	5 (10 %)	4 (9 %)	5 (10 %)	3 (10 %)
	Anonymous uses only	8 (15 %)	7 (14 %)	7 (16 %)	8 (16 %)	4 (13 %)
Technical concerns	Technical and procedural security arrangements	28 (53 %)	26 (52 %)	22 (51 %)	27 (55 %)	15 (48 %)
	How long data will be retained	9 (17 %)	9 (18 %)	7 (16 %)	9 (18 %)	6 (19 %)
	Inherent risks or limitations of security on mobile device/internet	19 (36 %)	18 (36 %)	14 (33 %)	19 (39 %)	11 (35 %)
	The use of cookies	42 (79 %)	39 (78 %)	33 (77 %)	38 (78 %)	25 (81 %)
User rights	Procedures for opting out of data sharing^b,c^	30 (61 %)	28 (56 %)	25 (58 %)	30 (61 %)	19 (61 %)
	Consequences of not providing or sharing data^a^	15 (31 %)	15 (30 %)	13 (30 %)	15 (31 %)	8 (26 %)
	Procedures for subject access requests^b,c^	14 (29 %)	14 (28 %)	10 (23 %)	14 (29 %)	9 (29 %)
	Procedures for editing data held by developers/third parties^b,c^	29 (59 %)	27 (54 %)	23 (53 %)	29 (59 %)	17 (55 %)
	Procedures for deleting data held by developers/third parties^b,c^	15 (31 %)	14 (28 %)	14 (33 %)	15 (31 %)	10 (32 %)
	Complaints procedures^c^	28 (53 %)	27 (54 %)	24 (56 %)	28 (57 %)	17 (55 %)
	Special procedures for handling data for vulnerable users	9 (17 %)	9 (18 %)	8 (19 %)	9 (18 %)	6 (19 %)
Administrative details	Identify data controller or responsible legal entity	16 (30 %)	16 (32 %)	14 (33 %)	16 (33 %)	10 (32 %)
	Legal jurisdiction governing policy	27 (51 %)	26 (52 %)	23 (53 %)	26 (53 %)	17 (55 %)
	Jurisdictions under which data will be processed^a^	13 (27 %)	13 (26 %)	11 (26 %)	13 (27 %)	8 (26 %)
	Date of policy	8 (15 %)	7 (14 %)	5 (12 %)	8 (16 %)	3 (10 %)
	Date of next review	0 (0 %)	0 (0 %)	0 (0 %)	0 (0 %)	0 (0 %)
	Procedures for changing the terms of the policy	17 (32 %)	17 (34 %)	14 (33 %)	17 (35 %)	11 (35 %)

^a^Incorporates strong personal identifiers, health-related information and other sensitive information; ^b^because these topics are only relevant for apps that transmit data, the denominator for calculated percentages is the number of apps with a privacy policy that also transmit data; ^c^for these domains, policies were additionally examined to distinguish between rights afforded to individuals and those denied. However, in no case did a policy text mention a user right only to deny it

### Concordance of policies and data handling practices

Overall, 71 % (n = 50/70) of apps collecting or transmitting information (or both) also had a privacy policy. For a small number (4 %, n = 2/49) information handling was completely consistent with commitments made by the policy. However, while no apps transmitted information where a specific commitment had been made not to, four-fifths either collected (82 %, n = 42/49) or transmitted (78 %, n = 38/49) one or more data items not addressed by a policy. Most commonly, collections consisted of personal identifiers (n = 35) or health-related information (n = 22) which would be obvious or expected. However, nearly half of apps did not fully disclose that strong personal identifiers (n = 47 %, 23/49) would be transmitted and a quarter of apps (24 %, n = 12/49) sent analytics information without informing users. Of the eight apps stating that data collection would be anonymous, seven displayed behaviour consistent with this claim, using either a randomly generated identifier (n = 6) and/or weaker identifiers (n = 4) to identify users. One app, however, used a fixed device identifier when reporting analytics data.

### Operating system permissions

The assessment approach used to evaluate permissions requested by apps to access operating system components, such as device cameras or contact databases, varied by platform. For apps available on Android (n = 42), a systematic assessment was performed against permissions explicitly declared at the time of app installation. Common permission requests for which justifiable uses existed in all cases were for network access by 93 % (n = 49/42), requesting device state by 71 % (n = 30/42) and accessing local data storage by 52 % (n = 22/42). Less common permissions for which justifiable purposes were also apparent were to enable a user to place a call by activating a number stored by an app (26 %, n = 11/42), provide notifications using vibration (17 %, n = 7/42), receive push notifications (5 %, n = 2/42) and, in one diary app, connect to a Bluetooth weighing scale. However, while almost two-fifths (38 %, n = 16/42) requested geolocation permissions, a clear reason, such as a mapping function, was identified in only five cases. Similarly, justifiable reasons were only found in one out of three apps requesting device camera access and two of three apps requesting access to a local contacts database, and one app requested calendar permissions without a clear purpose. In no case where permissions were requested without a clear use apparent in the app was there evidence of data being collected via that route, suggesting that these findings represent inaccurate permission declarations rather than malicious intent.

In contrast to Android, iOS apps (n = 37 not available on Android) used an on-demand approach to permission management, where an opportunity to grant or reject access was generated at the time when a particular feature was first used. Permission requests were identified, therefore, during the testing program. In general, no unexpected prompts were encountered. However, a small number of apps (n = 6) produced by a single developer, unexpectedly requested location information while displaying written information.

## Discussion

Medical app stores and accreditation programs are specifically intended to provide a trusted resource for both patients and clinical users. In addition to offering clinical quality assurances, they are well-placed to scrutinize information privacy. Some, including the NHS Health Apps Library, incorporate specific commitments to that effect [22, 34]. These are topical concerns for patients and the public. Almost two-thirds of US adults asked about the electronic exchange of medical information in clinical settings identified privacy as a salient issue [35]. Users can struggle to accurately gauge privacy risks in general app stores [36, 37] and often report feeling uncomfortable about information sharing practices in otherwise valued apps [38]. Unwanted disclosures of medical information prompt understandable concerns about impacts on relationships, health insurance and employment. Certification and curated app ‘whitelists’ offer a route to allay these worries. However, if assurances are offered, they must be robust.

Apps available through the NHS Health Apps Library exhibited substantial variation in compliance with data protection principles, demonstrated both by the availability and content of privacy policies, and adherence to recommended practices for confidentiality enforcement. Over half included functions in which personal details, health-related information, or both, were transferred to online services, but a fifth of such apps, and two-thirds of apps overall, did not have a privacy policy. In this respect, health apps, whether accredited or not [10], appear to be little better than non-medical apps available through general app stores [39], despite greater potential sensitivities surrounding health-related information. While most, but not all, privacy policies explained how information would be used, coverage of other aspects that would enable a user to make an informed choice about which information to disclose was less consistent. For example, a sixth of apps sent information to advertisers and third-party analytics but did not mention secondary uses of information in a policy. While there was no evidence of malicious intent, a fifth of apps shared limited information, including in some cases details of medical topics that users had viewed or search for, with advertising and marketing companies. Procedures enabling user rights afforded by data protection law, such as the ability to view and amend personal data, were inconsistently documented in privacy policies. The observed variation prompts questions about the coverage, and consistency, achieved by the certification process. For example, it was not clear why differences in the likelihood of having a privacy policy by payment model or platform should exist in apps available through a common accreditation framework.

Two cloud-based apps had critical privacy vulnerabilities; weaknesses of design that could be intentionally exploited to obtain user information. As long as these vulnerabilities persist, the privacy of users of these services is in jeopardy. As recent data thefts from high profile online services have shown, the risk is not simply theoretical [40, 41]. Many apps took inadequate steps to secure personal information, whether stored locally on devices or being transmitted to online services. Most concerning was the finding that some apps sent personal information without the use of encryption. Mobile communications may be particularly at risk of interception because, unlike fixed computers, information is sent using public computer networks for which users have little control over confidentiality enforcement arrangements. A small number of apps transmitted both unsecured personal and health information, for example research data pairing device and personal identifiers with details of substance use. However, the bigger potential risk to privacy is probably identity-related. Half of apps transmitting user account details sent usernames and passwords unencrypted. Armed with such information, a malicious user might be able to access other resources, for example email or online bank accounts. We found examples of complete personal datasets, including name, date of birth and contact details, sent as plain text. No apps encrypted local data stores, despite the widespread use of PIN or password security within apps that might reasonably lead a user to believe their information was protected. While recent changes proposed by operating system manufacturers aim to ensure that information stored on devices are encrypted by default, responsibility for ensuing confidentiality during transmission will remain with developers. A failure to implement appropriate technical safeguards of personal information does not only imply a failure of accreditation, it may also represent a violation of data protection law in the UK [23].

The findings highlight potential shortcomings of an accreditation approach that, in respect of privacy at least, appears to rely mainly on self-declared compliance. The strategy contrasts with that adopted by AppSaludable, another prominent certification program, which explicitly defines privacy criteria and combines structured self-assessment with a formal evaluation process [42]. To our knowledge, however, the guarantees offered by this program have not been subject to independent assessment. Recent work has highlighted the potential challenge of defining appropriate governance standards for fast-changing and diverse technologies like health apps [11, 43]. In addition to being comprehensive and consistent, governance arrangements need to be feasible, both for developers and those appraising apps. The release of apps in multiple jurisdictions potentially complicates the process of reconciling different privacy requirements [44]. The ‘surface area’ of potential privacy risks is large. Most apps included in this study exchanged information with multiple services, including developer and third-party data-hosting, analytics and content providers. Privacy issues are likely to only become more complex with the advent of wearable medical technologies, devices connected via the Internet of Things [45] and large-scale ‘big data’ analyses that combine information about individuals from multiple sources. User attitudes to, and expectations of, privacy are dynamic and sometimes contradictory [38], and appraisal will need to reflect changing norms. For example, today many users appear comfortable disclosing certain types of health-related information, such as weight loss or smoking cessation, on public social networks. In a health context, such information would still be considered unambiguously confidential, based on legislative frameworks that are, in some cases, almost two decades old [44]. Some issues are subtle, for example risks associated with hosting web pages, which may contain separate tracking code, within apps. However, many of the privacy issues we identified are well-recognized [6, 43], addressed by best-practice guidelines targeting developers [31, 46–48], and required little technical knowledge to uncover. Although exotic privacy vulnerabilities are anticipated for mobile health [6], all those identified in this study existed on a list of top-ten issues for mobile technologies [49]. Indeed, the greater threat appeared to be the risk of identity theft rather than compromise of medical information. At the very least, accreditation should ensure compliance with industry-standard levels of encryption and authentication.

The implications extend beyond the risk of diminished trust in health apps. Precarious privacy practices may create new legal and liability issues, and these may ultimately require regulator involvement. Because health and, in particular, wellness apps are often provided by organizations that are not traditional medical providers, they can sit outside the scope of existing legal and professional safeguards [50]. In addition to questions of information ownership, health apps raise the prospect of potentially sensitive information being processed by organizations with limited health experience, in jurisdictions with varying levels of information protection. Within healthcare organizations, existing governance structures provide a basis for managing app-related risks. However, these need to be able to adapt to the changing technical landscape, and there remain unresolved questions of liability [51]. Recently, regulators in both the USA and Europe have started to take action to address clinically unsafe and ineffective apps [52, 53]. Our findings suggest that privacy concerns should also routinely feature in discussions about future regulation of medical apps [51, 54], both as part of accreditation programs and in the wider marketplace. Discussion should also consider the balance between policy-level interventions and the role of technical strategies that might mitigate particular privacy risks. Recent developments include using ‘just-in-time’ strategies for alerting users to potential privacy risks, ‘blurring’ personal information to reduce its value to potential identity thieves and infrastructure designed to secure the online processing of clinical data [31, 44]. Viable solutions will need to scale to the growing number of health apps, be applicable across the variety of app platforms, and be acceptable to patients, developers and regulators.

By assessing all apps available through an accredited medical app store we were able to sample a wide range of app types including those from health providers and commercial organizations. The frequencies of identified issues reflect the specific population of apps available at the time of assessment. Interpretation should take account of the possibility that new and updated apps will exhibit different privacy-related characteristics. This does not affect the value of specific issues that need to be addressed, nor broader patterns existing within the data, for example inconsistencies in approaches to securing information. However, there is an ongoing requirement to ensure that new issues are identified and prioritized appropriately. The design of the study allowed us to examine local app behaviour and the content of transmissions originating from, and targeted towards, our test devices. However, we did not have access to information once received by either developer or third-party services, nor were we able to observe how data were handled at an organizational level by those services. Either or both of these may be sources of additional privacy risks not directly quantifiable by this study. These may arise as a result of technical and organizational challenges in ensuring the appropriate storage, handling and transfer of information held in online storage [55]. Our approach, instead, relied on the degree to which those practices were affirmed in a suitable privacy policy, which may be an imperfect proxy for actual behaviour. Recent work has illustrated the scope for threats arising from online storage of health information and identified privacy-preserving strategies that could inform future studies that assess compliance more directly [56].

## Conclusions

Variation in privacy practices observed in clinically-accredited health apps available through a dedicated medical app store raises concerns about potential risks to users and questions the ability of accreditation processes relying substantially on developer self-certification to ensure adherence to data protection principles. Regulators should consider establishing standards for accreditation processes, and be ready to intervene if accreditation programs cannot manage risks effectively. If patients or the public are deterred from using apps because of questions of trust, then the potential clinical benefits of mobile health will not be realized [57].

## Additional files

Additional file 1:
**NHS health apps and privacy.** **Table AF1**: Data types identified in app data collections and transmissions. **Figure AF2**: How a ‘man-in-the-middle’ attack can be extended to intercept secure network communication. **Figure AF3**: Screenshot of custom software used to review transmitted data. **Table AF4**: Coding schema used to assess the privacy and security-related content of policy documents. **Figure AF5**: Screenshot of custom software used to annotate policy text. **Table AF6**: Characteristics of excluded apps. **Table AF7**: Characteristics of included apps. **Table AF8**: Summary of data transmission. (PDF 908 kb) Additional file 2:
**NHS health apps and privacy: dataset.** (XLSX 1569 kb)

## Abbreviations

API: Application programming interface allows one software component for example, a mobile app, to execute functions in another, for example, a cloud-based online service, without special knowledge about how the second component operates. The interface defines the functions, the parameters required to execute them and the form of any data that are returned
HTTP: Hypertext Transfer Protocol the scheme by which web pages and associated content are transferred between a server to a web browser
HTTPS: Secure variant of HTTP
ICO: Information Commissioner’s Office
IMEI: International Mobile (Station) Equipment Identity a fixed identifier assigned to mobile phones
iOS: iPhone/iPad operating system
IP: Internet Protocol the main scheme governing the exchange of information between computers across the Internet. An IP address is a numerical identifier assigned to individual computers used to route information
MAC: Media access control (address) a unique fixed identifier assigned to a network interface, for example a router or mobile-device wireless card
NHS: National Health Service
PIN: Personal identification number a numeric password

## References

[1] Klasnja P, Pratt W (2011). Healthcare in the pocket: mapping the space of mobile-phone health interventions. J Biomed Inform..

[2] research2guidance. Mobile health market report 2013–2017 [http://www.research2guidance.com/shop/index.php/downloadable/download/sample/sample_id/262/]

[3] Comstock J. Survey: 32 percent of mobile device owners use fitness apps [http://mobihealthnews.com/29358/survey-32-percent-of-mobile-device-owners-use-fitness-apps/]

[4] Manhattan Research. 2014 environment [http://manhattanresearch.com/Products-and-Services/Physician/Taking-the-Pulse-U-S]

[5] Steinhubl SR, Muse ED, Topol EJ (2013). Can mobile health technologies transform health care?. JAMA..

[6] Kotz D (2011). A threat taxonomy for mHealth privacy. Third International Conference on Communication Systems and Networks (COMSNETS), 4–8 January 2011.

[7] Cohn SP (2006). Privacy and confidentiality in the nationwide health information network.

[8] Smith HJ, Dinev T, Xu H (2011). Information privacy research: an interdisciplinary review. MIS Q..

[9] Njie L. Mobile health and fitness apps: what are the privacy risks? [https://www.privacyrights.org/mobile-health-and-fitness-apps-what-are-privacy-risks]

[10] Sunyaev A, Dehling T, Taylor PL, Mandl KD (2014). Availability and quality of mobile health app privacy policies. J Am Med Inform Assoc..

[11] Dehling T, Gao F, Schneider S, Sunyaev A (2015). Exploring the far side of mobile health: information security and privacy of mobile health apps on iOS and Android. JMIR Mhealth Uhealth..

[12] He D, Naveed M, Gunter CA, Nahrstedt K (2014). Security concerns in Android mHealth Apps. AMIA 2014 Annual Symposium, 15–19 November 2014.

[13] Adhikari R, Richards D (2014). Security and privacy issues related to the use of mobile health apps. 25th Australasian Conference on Information Systems, 8–10 December 2014.

[14] NHS Choices. Health apps library – safe and trusted apps to help you manage your health [http://apps.nhs.uk/]

[15] Agencia de Calidad Sanitaria de Andalucia. [Distintivo appsaludable] [http://www.calidadappsalud.com/]

[16] England NHS (2014). Five year forward view.

[17] Happtique I. Happtique: recommend the best apps (iOS, Android) to patients [https://www.happtique.com/]

[18] MyHealthApps.net. My health apps – tried and tested by people like you [http://myhealthapps.net/]

[19] LaRose R, Rifon N (2006). Your privacy is assured - of being disturbed: websites with and without privacy seals. New Media & Soc..

[20] Dolan B. Happtique suspends mobile health app certification program [http://mobihealthnews.com/28165/happtique-suspends-mobile-health-app-certification-program/]

[21] Singh I. Introducing the health apps library [http://www.england.nhs.uk/2013/03/13/health-apps-blog/]

[22] NHS Health Apps Library. Review process [http://apps.nhs.uk/review-process]

[23] HM Government. Data Protection Act 1998 [http://www.legislation.gov.uk/ukpga/1998/29/contents]

[24] iFunBox Dev Team. iFunBox – app installer & file manager for iPhone, iPad and iPod Touch 2.7 [http://www.i-funbox.com/]

[25] ES APP Group. ES file explorer file manager 3.0.7.0 [https://play.google.com/store/apps/details?id=com.estrongs.android.pop]

[26] Open Web Application Security Project. Man-in-the-middle attack [https://www.owasp.org/index.php/Man-in-the-middle_attack]

[27] Cortesi A. mitmproxy: a man-in-the-middle proxy 0.9.2 [http://mitmproxy.org/]

[28] Callegati F, Cerroni W, Ramilli M (2009). Man-in-the-middle attack to the HTTPS protocol. IEEE Secur Priv..

[29] Open Web Application Security Project. Certificate and public key pinning [https://www.owasp.org/index.php/Certificate_and_Public_Key_Pinning]

[30] Information Commissioner’s Office (2010). Privacy notices code of practice.

[31] Information Commissioner’s Office. Privacy in mobile apps – guidance for app developers [http://ico.org.uk/for_organisations/data_protection/topic_guides/online/~/media/documents/library/Data_Protection/Detailed_specialist_guides/privacy-in-mobile-apps-dp-guidance.pdf]

[32] European Commission. Commission decisions on the adequacy of the protection of personal data in third countries [https://web.archive.org/web/20150628225441/http://ec.europa.eu/justice/data-protection/document/international-transfers/adequacy/index_en.htm]

[33] Open Web Application Security Project. SQL injection [https://www.owasp.org/index.php/SQL_Injection]

[34] Agencia de Calidad Sanitaria de Andalucia. [Estrategia de calidad y seguridad en aplicaciones móviles de salud – confidencialidad y privacidad] [http://www.calidadappsalud.com/recomendaciones/confidencialidad-privacidad/]

[35] Agaku IT, Adisa AO, Ayo-Yusuf OA, Connolly GN (2014). Concern about security and privacy, and perceived control over collection and use of health information are related to withholding of health information from healthcare providers. J Am Med Inform Assoc..

[36] King J (2012). “How come I’m allowing strangers to go through my phone?” – smartphones and privacy expectations. Symposium on Usable Privacy and Security (SOUPS), 11–13 July 2012.

[37] Boyles JL, Smith A, Madden M. Privacy and data management on mobile devices [http://www.pewinternet.org/2012/09/05/privacy-and-data-management-on-mobile-devices/]

[38] Shklovski I, Mainwaring SD, Skúladóttir HH, Borgthorsson H (2014). Leakiness and creepiness in app space: perceptions of privacy and mobile app use. 32nd annual ACM CHI Conference on Human Factors in Computing Systems, 26 April–1 May 2014.

[39] Office of the Privacy Commissioner of Canada. Results of the 2014 Global Privacy Enforcement Network sweep [https://www.priv.gc.ca/media/nr-c/2014/bg_140910_e.asp]

[40] Finkle J, Chatterjee S, Maan L. EBay asks 145 million users to change passwords after cyber attack [http://www.reuters.com/article/2014/05/21/us-ebay-password-idUSBREA4K0B420140521]

[41] Harris KD. California data breach report [http://oag.ca.gov/ecrime/databreach/reporting]

[42] Ferrero-Alvarez-Rementeria J, Santana-Lopez V, Escobar-Ubreva A, Vazquez-Vazquez M. Quality and safety strategy for mobile health applications: a certification programme. Eur J ePractice. 2013.

[43] Plachkinova M, Andres S, Chatterjee S (2015). A taxonomy of mHealth apps – security and privacy concerns. The 48th Hawaii International Conference on System Sciences (HICSS), 5–8 January 2015.

[44] Martinez-Perez B, de la Torre-Diez I, Lopez-Coronado M (2014). Privacy and security in mobile health apps: a review and recommendations. J Med Syst..

[45] Weber RH (2010). Internet of Things – new security and privacy challenges. Comput Law Secur Rev..

[46] Bureau of Consumer Protection. Marketing your mobile app: get it right from the start [http://www.business.ftc.gov/documents/bus81-marketing-your-mobile-app]

[47] Federal Trade Commission (2013). Mobile privacy disclosures – building trust through transparency.

[48] Organisation for Economic Co-operation and Development. The OECD privacy framework 2013 [http://www.oecd.org/sti/ieconomy/oecd_privacy_framework.pdf]

[49] OWASP Mobile Security Project. Top ten mobile risks – final list 2014 [https://www.owasp.org/index.php/Projects/OWASP_Mobile_Security_Project_-_Top_Ten_Mobile_Risks]

[50] Hall JL, McGraw D (2014). For telehealth to succeed, privacy and security risks must be identified and addressed. Health Aff (Millwood).

[51] Yang YT, Silverman RD (2014). Mobile health applications: the patchwork of legal and liability issues suggests strategies to improve oversight. Health Aff (Millwood).

[52] Federal Trade Commission. “Acne cure” mobile app marketers will drop baseless claims under FTC settlements [http://www.ftc.gov/news-events/press-releases/2011/09/acne-cure-mobile-app-marketers-will-drop-baseless-claims-under]

[53] Medicines and Healthcare Regulatory Agency. Guidance on medical device stand-alone software (including apps) [http://www.mhra.gov.uk/Howweregulate/Devices/Software/index.htm]

[54] Cortez NG, Cohen IG, Kesselheim AS (2014). FDA regulation of mobile health technologies. N Engl J Med..

[55] Takabi H, Joshi JBD, Gail-Joon A (2010). Security and privacy challenges in cloud computing environments. IEEE Secur Priv..

[56] Abbas A, Khan SU (2014). A review on the state-of-the-art privacy-preserving approaches in the e-Health clouds. IEEE J Biomed Health Inform..

[57] Huckvale K, Car J (2014). Implementation of mobile health tools. JAMA..

