# Supplementary material for: Unaddressed privacy risks in accredited health and wellness apps: a cross-sectional systematic assessment
Source: BMC Med. 2015 Sep 7;13:214. doi: 10.1186/s12916-015-0444-y (PMC4582624; doi:10.1186/s12916-015-0444-y)
Supplement: Additional file 1: — NHS health apps and privacy. Table AF1: Data types identified in app data collections and transmissions. Figure AF2: How a ‘man-in-the-middle’ attack can be extended to intercept secure network communication. Figure AF3: Screenshot of custom software used to review transmitted data. Table AF4: Coding schema used to assess the privacy and security-related content of policy documents. Figure AF5: Screenshot of custom software used to annotate policy text. Table AF6: Characteristics of excluded apps. Table AF7: Characteristics of included apps. Table AF8: Summary of data transmission. (PDF 908 kb) [file 12916_2015_444_MOESM1_ESM.pdf]

Table AF1

Data types identified in app data collections and transmissions

**Data type group**	**Data type**
Strong personal Identifiers – Personal information that alone may uniquely identify an individual or allow identity to be compromised.	Date of birth
	Email address
	Financial/Payment Details
	Fixed Device Identifier (e.g. IMEI Number, MAC Address)
	Full Name
	Full Postcode (Zip Code)
	Medical System Numbers (e.g. Insurer Number)
	Others Contact Details
	Photographs of People
	Postal Address
	Social Network Credentials
	Social Security Number
	Telephone Numbers
	Username and/or Password
Weaker Personal Identifiers – Personal information that may be combined or linked to other data to uniquely identify an individual or allow identity to be compromised.	Age or Year of Birth
	Arbitrary Unique Identifier (e.g. Pseudonymization Key)
	Country or Area or Partial Postcode (Zip Code)
	First Name
	Gender
	Geolocation Information
Health-Related Information – Sensitive personal information relating to health status and medical history.	Disability Status
	Genetic Information
	HIV Status
	IVF Information
	Measured Parameters (e.g. Weight, Blood Glucose)
	Medical History
	Medications
	Mental Health Status
	Substance Use
Other Sensitive Information – Sensitive personal information not covered in Health-Related	Employment Status
	Ethnicity
	Political Affiliation

Data type group	Data type
Information.	Religious Beliefs
	Sexuality
Other User-Generated Data – personal information not covered in categories above.	Hobbies and Interests
	User-Generated Content Not Otherwise Covered
Analytics Data	Usage Data
Protocol-Standard Data [a]	Device Characteristics (e.g. User-Agent Header)
	IP Address

[a] Data sent routinely as part of network communications over which app has limited control.

Figure AF2

How a "man-in-the-middle" attack can be extended to intercept secure network communication

Secure communication commonly involves the exchange of certificates that verify identify and allow data to be encrypted before sending across a network. By intercepting the initial secure connection setup process (1) and impersonating the mobile device's identity (2), an intercepting computer can gain access to a legitimate certificate (3) while issuing its own, bogus certificate (4). Once hijacked, an attacker can both read encrypted outgoing traffic (5) as well as any incoming data (6). In a real-world setting, configuration settings on the user device must typically be altered so that it does not reject the bogus certificate. Technical details have been simplified to communicate key principles.

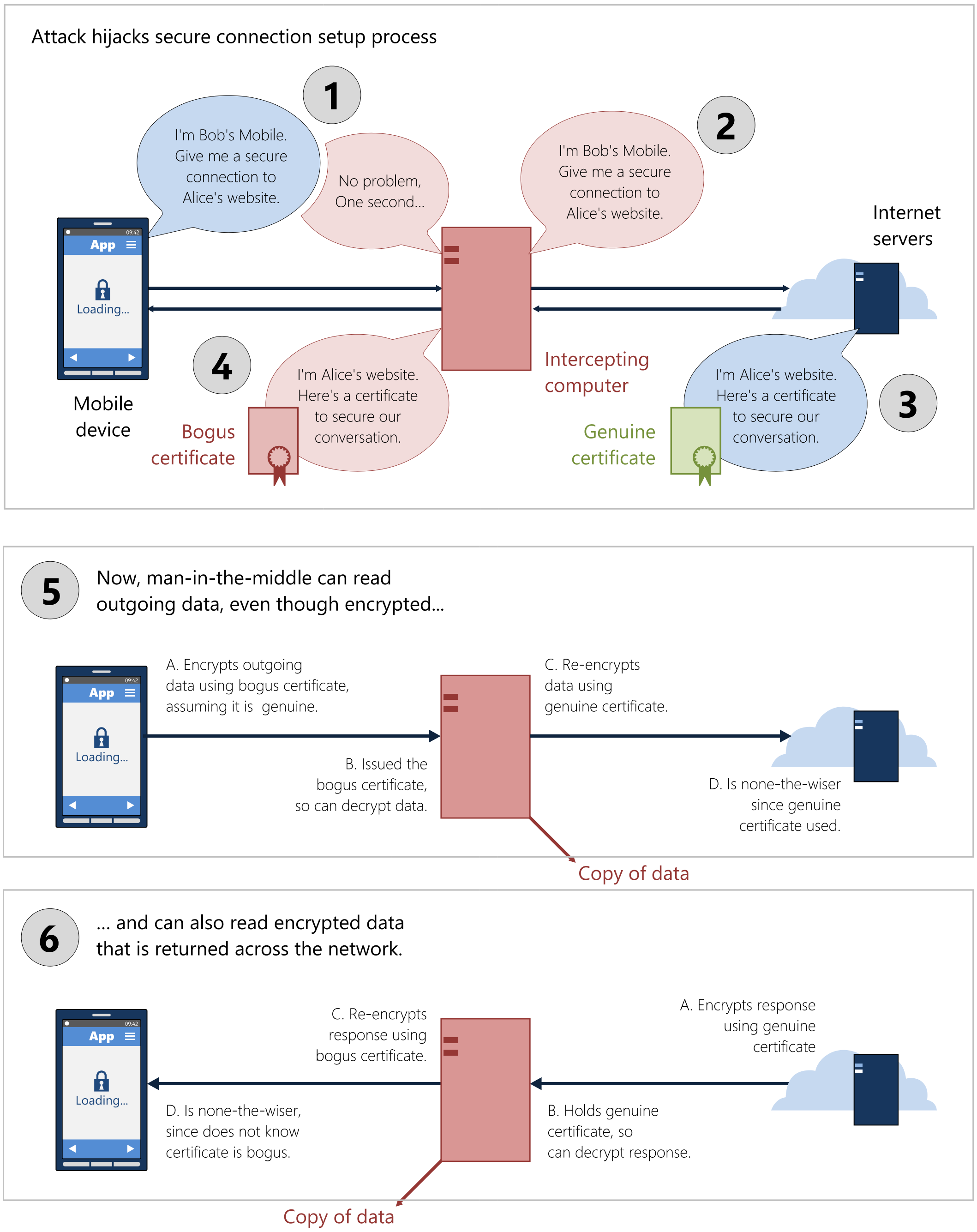

Figure AF3

Screenshot of custom software used to review transmitted data

Software reconciled outgoing requests generated by apps and the responses received from Internet-based servers, and allowed the content of these paired request-response messages to be inspected. The overall purpose of the message as well as transmission of specific data types could then be annotated using the tool. In the toy example shown, transmission of a user email address to a cloud service provided by the app developer has been identified in an encrypted message.

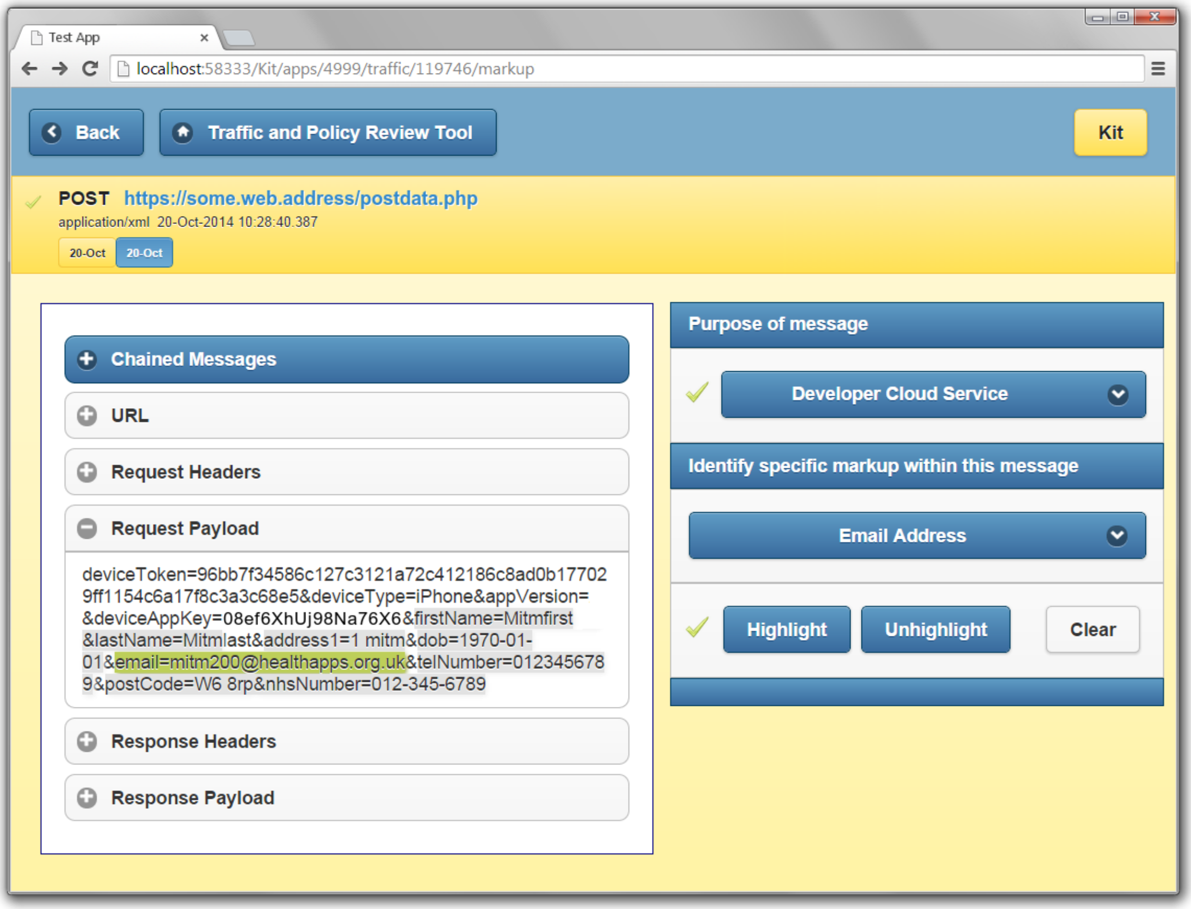

Table AF4

Coding schema used to assess the privacy and security-related content of policy documents

Domain	Topic
Uses of data	• Primary uses of collected data (e.g. administering accounts, contacting users, providing and improving services)<br>• Secondary uses of collected data (e.g. repackaging data for research or marketing purposes, mandatory disclosures)<br>• Sending data to developer-provided online services (e.g. online databases)<br>• Sending data to advertisers/marketers<br>• Sending data for analytics/research<br>• Sending data while loading content (e.g. satisfying search requests)<br>• Anonymous uses only
Technical concerns	• Technical and procedural security arrangements (e.g. anonymization, encrypted data transport, secure servers, limited access, backup)<br>• How long data will be retained<br>• Inherent risks or limitations of security on mobile device/Internet<br>• The use of cookies
User rights	• Procedures for opting out of data sharing<br>• Consequences of not providing or sharing data<br>• Procedures for subject access requests<br>• Editing and deleting data held by developers/third parties<br>• Complaints procedures<br>• Special procedures for handling data for vulnerable users and minors
Administrative details	• Identify of data controller or responsible legal entity<br>• Legal jurisdiction governing policy<br>• Jurisdictions under which transmitted data will be processed<br>• Date of policy<br>• Date of next review<br>• Procedures for changing the terms of the policy

Figure AF5

Screenshot of custom software used to annotate policy text

Software supported a step-by-step process of review for each policy document to identify whether specific aspects of the policy coding schema were addressed. Relevant policy text could be selected and annotated. In the example shown, text relating to the transmission of data for analytics purposes has been identified.

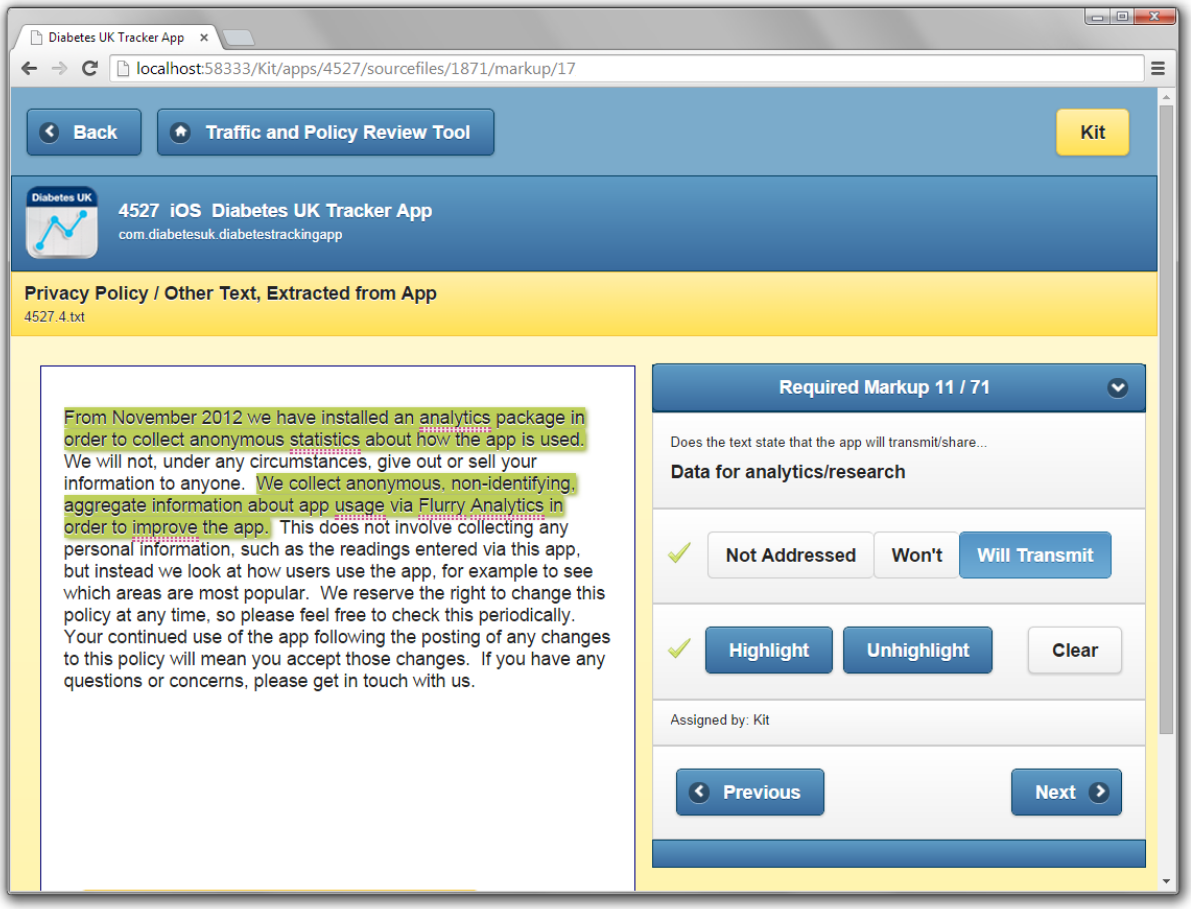

Table AF6

Characteristics of excluded apps

App Name	Available for iOS platform?	Available for Android platform?	Developer	Cost	Reason for Exclusion
eRedbook	Yes (0.35)	No	Sitekit Solutions Ltd	Free	Unable to log in.
FolUp for Patient	Yes (1.0)	Yes (1.0)	Mobilelite Ltd	Free	Not available for download (iOS). Unable to log in (Android).
FoodWiz	Yes (1.3)	Yes (1.5)	Food Angels UK Ltd.	Free	Unable to log in (iOS). Would not start (Android).
Health Fabric	Yes (Unknown)	Yes (Unknown)	Sensory Software International	Free	Not available for download (Android). Unable to log in (iOS).
HIV Test Finder	Yes (2.1)	No	Thomas Paterson	Free	Duplicate of Aidsmap News.
mproAutism	Yes (1.0.3)	Yes (1.0.3)	Crimson Tide Mpro Limited	Free	Unable to log in.
Predictable	Yes (3.3)	No	Therapy Box Limited	178.18 USD	App cost (£109.99) exceeds threshold.
Stable Angina Patient Decision Aid	~~No~~ Yes (1.7) [a]	Yes (1.7)	Totally Health	Free	Not compatible with any test device (Android).
Talking Point	~~No~~ Yes (105) [a]	Yes (2.4.8.3)	Alzheimer's Society	Free	Would not start (Android).
The Linden Method	Yes (2.0)	No	Lifewise Publishing Ltd	90.49 USD	App cost (£55.86) exceeds threshold.
Unity Core	Yes (1.0.1)	No	Liberator Ltd	153.88	Duplicate of Unity

App Name	Available for iOS platform?	Available for Android platform?	Developer	Cost	Reason for Exclusion
				USD	Core Lite. App cost (£94.99) exceeds threshold.

[a] iOS versions of these apps were not affected by installation or start-up problems and were included in the study. They are not included in the count of excluded apps, therefore.

Table AF7

Characteristics of included apps

App Name	Available for iOS platform?	Available for Android platform?	Developer	Cost	App Features
Aidsmap News	Yes (2.1)	No	Thomas Paterson	Free	Information
Antifungal Interactions	Yes (1.12)	Yes (1.0)	Graham Atherton	4.84 USD	Information
British Heart Foundation Recipe Finder	Yes (2.0)	Yes (1.0)	Precedent	Free	Information
Brush DJ	Yes (1.5)	Yes (1.6)	Benjamin Underwood	Free	Utility Function (Tooth Brushing Timer), Reminders
BSU Health	Yes (2.1)	Yes (1.06)	NC Bath Ltd	Free	Information, Health Promotion (Smoking, Alcohol, Drugs, Sexual Health), Service Directory
Calorie Counter +	Yes (2.4.1)	Yes (2.2.5)	NutraTech Ltd	Free	Health Promotion (Exercise/Weight Loss), Diary/Personal Health Record
Cancer Emergency Response Tool – CERT	No	Yes (1.01)	Dorset Cancer Centre	Free	Self-Management (Cancer), Self-Assessment, Symptom Checker, Therapy Management
CarePair	Yes (1.0)	No	Russell Smith	Free	Social Network
Change4Life drinks tracker	Yes (1.6)	Yes (1.4)	NHS Choices	Free	Health Promotion (Alcohol), Research Project, Information

App Name	Available for iOS platform?	Available for Android platform?	Developer	Cost	App Features
Change4Life Fun Generator	Yes (1.0)	Yes (1.0)	NHS Choices	Free	Health Promotion (Exercise/Weight Loss), Information
Change4Life Healthier Recipes	Yes (1.4)	Yes (1.2)	NHS Choices	Free	Health Promotion (Healthy Eating), Research Project, Information
Coronary Angiogram eSupport for patients	Yes (1.0.0)	No	Norton-Bates	4.84 USD	Information, Diary/Personal Health Record, Therapy Management
Dentify	Yes (1.0)	Yes (1.0)	Yatisha Patel	1.6 USD	Information
Depression Calculator	Yes (1.0)	No	Patient.co.uk	Free	Self-Assessment, Symptom Checker (Depression)
Diabetes Manager	Yes (1.0)	No	Patient.co.uk	Free	Self-Management (Diabetes), Diary/Personal Health Record, Information
Diabetes Risk Checker	Yes (1.0)	Yes (1.0)	Click Innovate Ltd	1.6 USD	Self-Assessment, Risk Checker (Diabetes)
Diabetes UK Tracker App	Yes (331)	Yes (1.4)	Diabetes UK	Free	Self-Management (Diabetes), Diary/Personal Health Record
Dr iSeb	Yes (1.0)	No	Dr iSeb limited	Free	Information
DrinkCoach	Yes (1.4)	No	Haringey Advisory Group on Alcohol (HAGA)	Free	Health Promotion (Alcohol), Diary/Personal Health Record

App Name	Available for iOS platform?	Available for Android platform?	Developer	Cost	App Features
Drinks Meter	Yes (1.2)	Yes (1.3)	Global Drug Survey	Free	Health Promotion (Alcohol, Drugs), Research Project, Self-Assessment, Risk Checker (Alcohol)
Epilepsy Toolkit	Yes (4.3)	No	MCM Net Limited	Free	Information, Self-Management (Epilepsy)
Fairfield Park Health Centre	Yes (1.3)	Yes (1.1)	NC Bath Ltd	Free	Information, Health Promotion (Smoking, Alcohol, Drugs, Sexual Health), Service Directory
Find NHS Services near you	No	Yes (0.2)	Smart Droid	Free	Information, Service Directory
Finerday	No	Yes (1.2)	Mobilelite Ltd	Free	Social Network
Gallstones eSupport for Patients	Yes (1.0.0)	No	Norton-Bates	4.84 USD	Information, Diary/Personal Health Record, Therapy Management
Gastric Band - eSupport	Yes (1.0.3)	No	Norton-Bates	3.22 USD	Information, Diary/Personal Health Record, Therapy Management
Gastric Bypass - eSupport	Yes (1.0.3)	No	Norton-Bates	3.22 USD	Information, Diary/Personal Health Record, Therapy Management
Gastric Sleeve -	Yes (1.0.3)	No	Norton-Bates	3.22	Information,

App Name	Available for iOS platform?	Available for Android platform?	Developer	Cost	App Features
eSupport				USD	Diary/Personal Health Record, Therapy Management
Grid Player	Yes (1.5.0.0)	No	Sensory Software International	Free	Assistive Technology
Hearts and Minds	No	Yes (1.2)	Garwood Medical	1.12 USD	Self-Assessment, Risk Checker (Cardiovascular Disease)
howRU Health Tracker	Yes (1.4.0)	No	Routine Health Outcomes Ltd	Free	Diary/Personal Health Record
iBreastCheck	Yes (8)	Yes (1.0.3)	Breakthrough Breast Cancer	Free	Information, Self-Assessment, Risk Checker (Breast Cancer Risk)
Isabel Symptom Checker	Yes (1.1)	Yes (1.0)	Isabel Healthcare	Free	Self-Assessment, Symptom Checker (Differential Diagnosis)
iSightTest	Yes (1.6)	No	Kay Pictures Ltd	34 USD	Self-Assessment (Eyesight)
Kent C Card	Yes (1.1)	Yes (1.2)	Kent Community Health NHS Trust	Free	Information, Health Promotion (Sexual Health), Service Directory
King St and University Practice Lancaster	Yes (1.4)	Yes (1.07)	NC Bath Ltd	Free	Information, Health Promotion (Smoking, Alcohol, Drugs, Sexual Health), Service Directory

**App Name**	**Available for iOS platform?**	**Available for Android platform?**	**Developer**	**Cost**	**App Features**
Knee Athroscopy -eSupport	Yes (1.0.0)	No	Norton-Bates	4.84 USD	Information, Diary/Personal Health Record, Therapy Management
Lab Tests Online UK	Yes (1.0.0)	Yes (1.0.00)	ACB	Free	Information
LIFESAVER	Yes (1.0.0)	Yes (1.01)	Unit9	Free	Information, First Aid Training
Lymphoedema Breast Cancer App	Yes (1.0.0)	Yes (1.0)	Kelly Foote	3.22 USD	Information, Self-Management (Lymphedema), Diary/Personal Health Record, Therapy Management
Me and Mine Health	Yes (1.0)	No	App Physio	2.11 USD	Self-Management (General), Diary/Personal Health Record
Me, Myself, and I	Yes (1.5)	No	Serious Games International	16.18 USD	Game
Medimapp	Yes (2026)	No	Medimapp Limited	Free	Information, Service Directory
Meningitis Signs and Symptoms	Yes (1.0.1)	Yes (1.0)	Meningitis Trust	Free	Information, Game
MyChoicePad Lite	Yes (943)	No	Insane Logic Ltd.	Free	Assistive Technology
NHS 24 MSK Help	Yes (1.6)	Yes (1.7)	NHS 24	Free	Information, Self-Management (Musculoskeletal Problems)

App Name	Available for iOS platform?	Available for Android platform?	Developer	Cost	App Features
NHS BMI healthy weight calculator and tracker	Yes (1.3)	No	NHS Choices	Free	Health Promotion (Exercise/Weight Loss), Diary/Personal Health Record, Research Project
NHS Drinks Tracker	Yes (1.0)	No	NHS Choices	Free	Health Promotion (Alcohol), Diary/Personal Health Record, Research Project
NHS Health and Symptom Checkers	Yes (2.0.1)	Yes (2.0.1)	NHS Direct	Free	Information, Self-Assessment, Symptom Checker (Differential Diagnosis)
NHS Quit Smoking	Yes (1.3)	No	NHS Choices	Free	Health Promotion (Smoking), Information
NTW – Northumberland Tyne and Wear NHS Foundation Trust	Yes (1.0.3)	Yes (1.0.9)	Northumberland, Tyne and Wear NHS Foundation Trust	Free	Information
Numberhood	Yes (1.4)	No	OCSI	Free	Information
OATBook	Yes (2.3)	No	Rob Cleaton	4.84 USD	Self-Management (Medication), Therapy Management
Panic Attack Aid	Yes (1.0)	Yes (1.0)	Panic Attack Aid	8.08 USD	Self-Management (Panic Attacks), Information, Self-

App Name	Available for iOS platform?	Available for Android platform?	Developer	Cost	App Features
					Management Tool
Parkinson's UK EasyCall	Yes (1.2.0.2)	Yes (2.0.0)	Parkinson's UK	Free	Assistive Technology, Self-Management (Parkinson's Disease)
Patient IBS	Yes (1.0.1)	Yes (1.0)	Patient.co.uk	3.22 USD	Self-Management (IBS), Diary/Personal Health Record, Information
Patient.co.uk	Yes (3.0)	Yes (2.1)	Patient.co.uk	Free	Information
PillManager	Yes (2.0.2)	Yes (2.1.1)	Healthnet Limited	Free	Self-Management (Medication, Diabetes, Hypertension), Diary/Personal Health Record, Service Directory, Pharmacy Services, Reminders
Rally Round	Yes (1.3)	No	Health2Works Ltd	Free	Social Network
RCP Stroke Guideline	Yes (2.0)	Yes (1.0)	Cranworth Medical Ltd	Free	Information, Self-Management (Stroke)
SiKL	Yes (2023)	No	NULL	Free	Self-Management (Sickle Cell Anemia)
Sleep Diary	Yes (1.2)	No	Patient.co.uk	Free	Diary/Personal Health Record, Health Promotion (Sleep), Information
Smoke Free	Yes (1.3)	No	David Crane	Free	Health Promotion (Smoking), Research Project, Diary/Personal Health

App Name	Available for iOS platform?	Available for Android platform?	Developer	Cost	App Features
					Record, Information
Smoking Time Machine	Yes (1.0.3)	Yes (1.0.2)	Rancon	1.6 USD	Heath Promotion (Smoking)
Stable Angina Patient Decision Aid	Yes (1.7)	~~No~~ Yes (1.7) [a]	Totally Health Ltd.	Free	Information
Stomawise Travel Certificate	Yes (1.1.0)	Yes (1.1.0)	John Walsh	Free	Utility Function (Multilingual Stoma Information), Self-Management (Stoma), Therapy Management
Talking Point	Yes (105)	~~No~~ Yes (2.4.8.3) [a]	Alzheimer's Society	Free	Social Network
Total Baby	Yes (3.1.2)	No	ANDESigned	8.08 USD	Diary/Personal Health Record, Reminders
Type 1 diabetes friend: alcohol guide	Yes (1.0)	Yes (1.11)	AP Apps	Free	Information
Unity Core Lite	Yes (1.0.1)	No	Liberator Ltd	Free	Assistive Technology
Weight Loss Surgery Scotland	Yes (2.1)	No	Richard Brady	Free	Information, Diary/Personal Health Record, Therapy Management
Weight Tracker	Yes (1.1)	Yes (1.0)	Patient.co.uk	Free	Health Promotion (Exercise/Weight Loss), Diary/Personal Health Record
Weightplan	Yes (2.5.2)	No	Weightplan	Free	Health Promotion

App Name	Available for iOS platform?	Available for Android platform?	Developer	Cost	App Features
			Limited		(Exercise/Weight Loss), Diary/Personal Health Record, Information
Welcome to St George's Hospital	Yes (1.2)	No	St George's Healthcare NHS Trust	Free	Health Promotion (Exercise/Weight Loss), Diary/Personal Health Record, Information
WellHappy	Yes (1.2.1364468092)	Yes (4)	NHS London	Free	Health Promotion (Sexual Health, Alcohol, Drugs)
Wellnote	Yes (3.1)	No	Wellnote	Free	Information, Service Directory, Diary/Personal Health Record, Self-Management (Diabetes, Hypertension, Medication), Reminders
WheelMate	Yes (1.0.1)	Yes (1.0.2)	Coloplast	Free	Information, Service Directory
Zombies, Run!	Yes (77)	Yes (2.2.0)	Six to Start	6.46 USD	Health Promotion (Exercise/Weight Loss), Diary/Personal Health Record
Zombies, Run! 5k Training	Yes (62)	Yes (1.1)	Six to Start	3.22 USD	Health Promotion (Exercise/Weight Loss), Diary/Personal Health Record

[a] Android versions of these apps could not be installed or would not start and were excluded.

Table AF8

Summary of data transmissions

Transmission destination and purpose	Apps transmitting data, n=70 (%)	Destination outside UK (% of apps transmitting data)
Developer-controlled services	23 (33%)	78 (305%)
Loading content	12 (17%)	4 (33%)-
Account-based services	7 (10%)	2 (29%)-
Crowd-sourced feedback	5 (7%)	2 (40%)-
Research data collection	5 (7%)	1 (20%)-
Third parties	63 (90%)	50 (79%)
Loading content	53 (76%)	17 (32%)
Account-based services	2 (10%)	1 (50%)
Crowd-sourced feedback	1 (1%)	0 (0%)
Marketing or advertising	14 (20%)	9 (64%)
Analytics data collection	43 (61%)	42 (98%)
Research data collection	1 (1%)	1 (10000%)
